# Extracellular Prion Protein Aggregates in Nine Gerstmann–Sträussler–Scheinker Syndrome Subjects with Mutation P102L: A Micromorphological Study and Comparison with Literature Data

**DOI:** 10.3390/ijms222413303

**Published:** 2021-12-10

**Authors:** Nikol Jankovska, Radoslav Matej, Tomas Olejar

**Affiliations:** 1 Department of Pathology and Molecular Medicine, Third Faculty of Medicine, Charles University and Thomayer University Hospital, 14059 Prague, Czech Republic; nikol.jankovska@ftn.cz (N.J.); radoslav.matej@ftn.cz (R.M.); 2 Department of Pathology, First Faculty of Medicine, Charles University and General University Hospital, 12800 Prague, Czech Republic; 3 Department of Pathology, Third Faculty of Medicine, Charles University and University Hospital Kralovske Vinohrady, 10034 Prague, Czech Republic

**Keywords:** Gerstmann–Sträussler–Scheinker syndrome, PrP, plaques, co-expression

## Abstract

Gerstmann–Sträussler–Scheinker syndrome (GSS) is a hereditary neurodegenerative disease characterized by extracellular aggregations of pathological prion protein (PrP) forming characteristic plaques. Our study aimed to evaluate the micromorphology and protein composition of these plaques in relation to age, disease duration, and co-expression of other pathogenic proteins related to other neurodegenerations. Hippocampal regions of nine clinically, neuropathologically, and genetically confirmed GSS subjects were investigated using immunohistochemistry and multichannel confocal fluorescent microscopy. Most pathognomic prion protein plaques were small (2–10 µm), condensed, globous, and did not contain any of the other investigated proteinaceous components, particularly dystrophic neurites. Equally rare (in two cases out of nine) were plaques over 50 µm having predominantly fibrillar structure and exhibit the presence of dystrophic neuritic structures; in one case, the plaques also included bulbous dystrophic neurites. Co-expression with hyperphosphorylated protein tau protein or amyloid beta-peptide (Aβ) in GSS PrP plaques is generally a rare observation, even in cases with comorbid neuropathology. The dominant picture of the GSS brain is small, condensed plaques, often multicentric, while presence of dystrophic neuritic changes accumulating hyperphosphorylated protein tau or Aβ in the PrP plaques are rare and, thus, their presence probably constitutes a trivial observation without any relationship to GSS development and progression.

## 1. Introduction

Intracellular or extracellular protein aggregates are characteristic hallmarks of neurodegenerative diseases [1]. Prion protein (PrP) and amyloid beta-peptide (Aβ) are extracellular amyloid protein deposits with a similar micromorphology that have been observed in prion diseases and Alzheimer’s disease (AD) [2]. Depending on the particular type of disease, extracellular deposits in prion disorders range from diffuse synaptic positivity to patchy/perivacuolar depositions to plaque-like deposits. In these, plaques can be subdivided into “daisy” plaques in kuru and kuru-like plaques, either solitary or multicentric [2]. In GSS, PrP plaques are composed of fibrils arranged in β-sheet secondary structure [3]. In PrP plaques, a certain level of co-expression with other amyloid-forming proteins can be observed in comorbidity, particularly in Alzheimer’s disease, where co-expression with Aβ plaques as well as with dystrophic neurites were previously observed [4].

Gerstmann–Sträussler–Scheinker syndrome (GSS—OMIM 137440) is a rare, slowly progressive prion disease caused by pathogenic mutations in the prion protein gene (PRNP). GSS is neuropathologically characterized by spongiform encephalopathy in different brain regions with varying severity and PrP-immunoreactive insoluble deposits mainly in the cerebral and cerebellar cortices and the basal ganglia. The most frequent mutation in GSS is in the PRNP gene (i.e., P102L); however, other mutations have been described in the literature [2]. Despite the different clinical symptomatology of the four recognized GSS P102L subtypes [5], there are no specific neuropathological changes that characterize the clinical subtypes. However, there is little data regarding the micromorphology of PrP deposits in GSS.

GSS can appear as a solitary disease; however, in some cases, comorbid neurodegenerations or comorbid neuropathology can exist, especially with hippocampal region involvement. In most cases, these are Alzheimer-related changes or so-called age-related deposits of hyperphosphorylated tau protein or protein TDP-43 [6].

Certain authors have reported co-expression of Aβ in the plaques of some GSS patients [7,8], while other studies noted the presence of hyperphosphorylated protein tau deposits [9] with three- or four-repeat tau (RD3 or RD4) proteins within PrP plaques [9,10].

Our study aimed to evaluate the micromorphology and protein composition of the hippocampal regions of archival brain material from nine confirmed GSS patients with pathogenic P102L PRNP mutations with regard to Aβ (a protein co-aggregate commonly observed in prion deposits) and hyperphosphorylated protein tau (as a marker of neuritic dystrophy). Moreover, we compared our results relative to the age of onset, disease duration, and methionine/valine (*M/V*) polymorphism at codon 129 of the PRNP gene in our cohort. We also examined data from other GSS cases published in the literature that contained information regarding micromorphology and the expression of pathological proteins.

## 2. Results

### 2.1. Immunohistochemistry

#### 2.1.1. PrP

**Figure 1:**
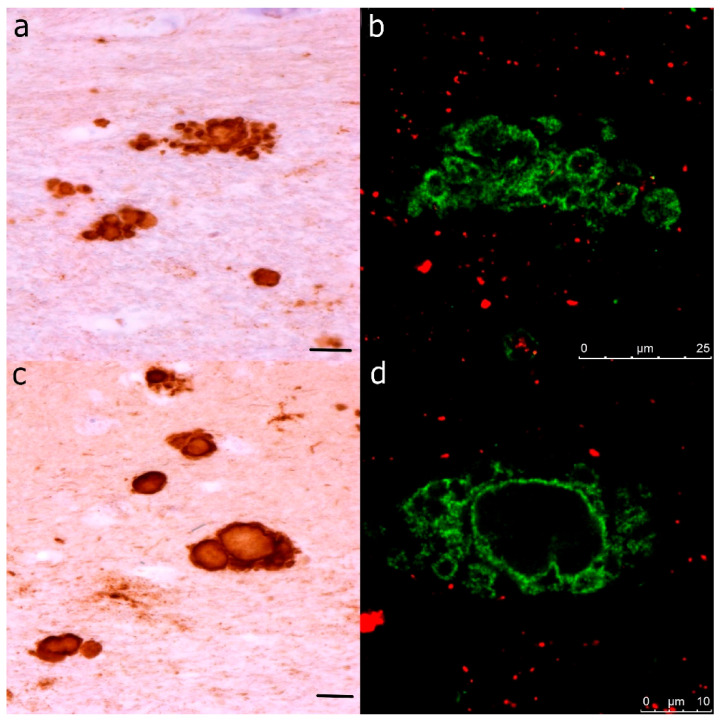
Illustration—Parallel observation of (**a**,**b**) multicentric and (**c**,**d**) solitary kuru-like plaques with centrally bright cores visualized using immunohistochemistry and immunofluorescence. (**a**,**c**) Primary antibody in immunohistochemical images: PrP (mouse monoclonal antibody). The secondary antibody was conjugated with horseradish peroxidase staining DAB. The original magnification was 100×. (**b**,**d**) Primary antibodies in immunofluorescent images: PrP (rabbit recombinant monoclonal antibody, green color) + AT8 (mouse monoclonal antibody, red color). The secondary antibody was conjugated with either Alexa Fluor^®^ 488 (anti-rabbit IgG, green) or Alexa Fluor^®^ 568 (anti-mouse IgG, red). Scale bars indicate 25 µm in (**a**,**b**) and 10 µm in (**c**,**d**).

**Figure 2:**
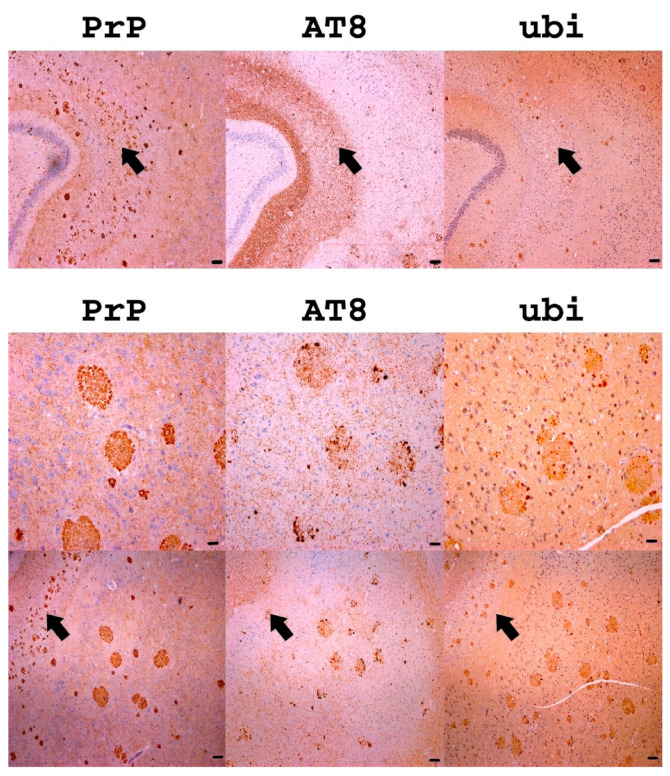
The top half—Immunohistochemistry observation of PrP, hyperphosphorylated protein tau, and ubiquitin in the CA1–CA2 area. No expression of hyperphosphorylated protein tau (AT8 antibody clone) and negligible ubiquitin expression was observed in areas with abundant small PrP aggregates. The magnification is 40× and the scale bars indicate 100 µm. The bottom half—Immunohistochemistry observation of PrP, hyperphosphorylated protein tau, and ubiquitin in large plaques in CA1. Bulbous changes in dystrophic neurites stained by AT8 and ubiquitin antibody were observed, but only in large PrP plaques of one particular subject; there was no co-expression in other areas (see arrows). All images come from subject no. 7. The images in the top row are zoomed details from the bottom row. The scale bars in the top row indicate 20 µm. The scale bars in the bottom row indicate 100 µm. Magnification is 100× and 40×, respectively.

Either diffuse, patchy/perivacuolar, and plaque positivity, containing kuru-like plaques including multicentric plaques containing pathological PrP was confirmed (using two different antibodies (clones 12F10 and 6H4)) in the hippocampal area of all nine subjects in the cohort. In the immunohistochemical staining, the kuru-like plaques appeared small to medium-sized (approximately 2–10 µm) spheroids with central brightness and were either dispersed solitary or in aggregates called multicentric plaques (illustrated in Figure 1). In two subjects (cases 4 and 7), large plaques, up to 100 µm, were observed in hippocampal area CA1 and the subiculum (see the bottom half of Figure 2).

#### 2.1.2. AT8 and Ubiquitin

Except for one subject, and irrespective of the expression of AD-related changes or hyperphosphorylated tau protein deposits in the form of primary age-related tauopathy (PART) and argyrophilic grain disease (AGD), there was generally no co-aggregation with hyperphosphorylated protein tau or ubiquitin in areas where PrP plaques were observed. In one subject (case 7), no co-aggregation of PrP with hyperphosphorylated protein tau was observed in areas of small solitary or multicentric plaques; a few plaques, which were slightly positive for ubiquitin, were found (top half of Figure 2). However, this subject also showed a significant co-aggregation of PrP with bulbous (extremely dilated) [11] dystrophic neurites staining for either hyperphosphorylated protein tau or ubiquitin in the same CA1 hippocampal area and the subiculum (see the bottom half of Figure 2).

### 2.2. Immunofluorescence

**Figure 3:**
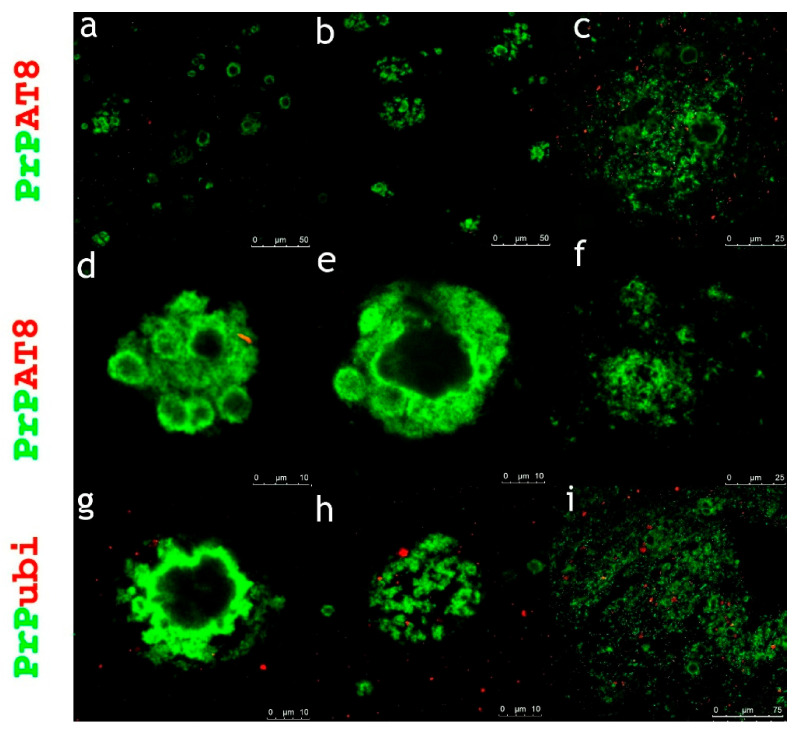
Condensed PrP aggregates as the dominant observation in GSS subjects. The dominant finding across the cohort was no or negligible co-aggregates with hyperphosphorylated protein tau and ubiquitin. Primary antibodies: PrP (rabbit recombinant monoclonal antibody, green color) + AT8 (mouse monoclonal antibody, red color), ubiquitin (mouse monoclonal antibody, red color). The secondary antibody was conjugated with either Alexa Fluor^®^ 488 (anti-rabbit IgG, green) or Alexa Fluor^®^ 568 (anti-mouse IgG, red). Scale bars indicate 50 µm in (**a**,**b**), 25 µm in (**c**,**f**), 10 µm in (**d**,**e**,**g**,**h**), and 75 µm in (**i**).

The dominant finding from laser scanning multichannel immunofluorescence microscopy in all subjects investigated was the presence of condensed PrP aggregates, either diffuse or in the form of plaques or kuru-like plaques without any significant tau or ubiquitin co-pathology (Figure 3). Immunofluorescence confirmed that kuru-like plaques were spheroids with centrally bright cores that were either solitary or multicentric (Figure 1).

**Figure 4:**
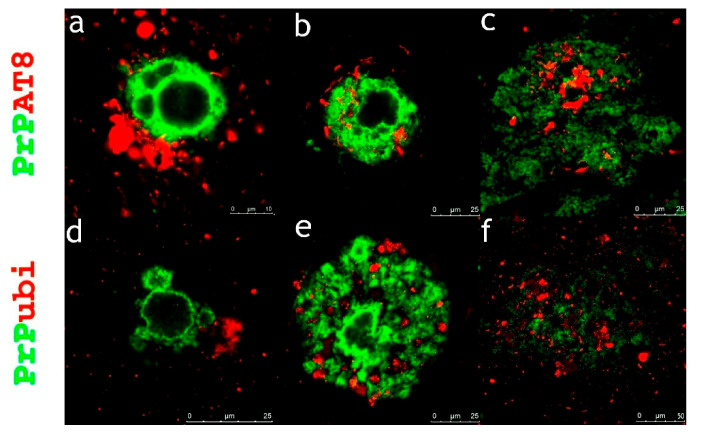
Few observations found condensed PrP with co-aggregates in GSS subjects. Across the cohort, few PrP aggregates either in plaques, kuru-like plaques, or diffuse exhibited a certain level of the hyperphosphorylated protein tau and ubiquitin co-pathology. Primary antibodies: PrP (rabbit recombinant monoclonal antibody, green color), AT8 (mouse monoclonal antibody, red color), ubiquitin (mouse monoclonal antibody, red color). The secondary antibody was conjugated with either Alexa Fluor^®^ 488 (anti-rabbit IgG, green) or Alexa Fluor^®^ 568 (anti-mouse IgG, red). Scale bars indicate 10 µm in (**a**), 25 µm in (**b**–**e**), and 50 µm in (**f**).

In a very few cases of plaques with diffuse deposits across the cohort of subjects investigated, dystrophic, less than more dilated neurites; however, accumulation of hyperphosphorylated protein tau or ubiquitin were observed (see Figure 4).

**Figure 5:**
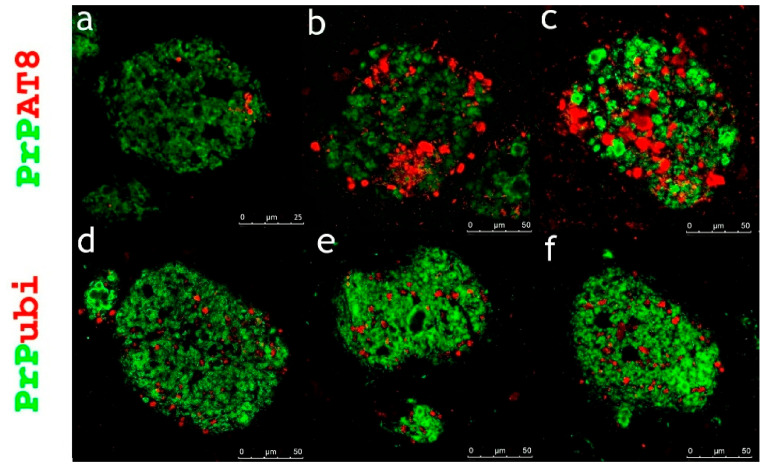
AT8 positive bulbous changes within huge diffuse PrP plaques—Immunofluorescence observation of co-expression PrP with hyperphosphorylated protein tau and ubiquitin in large plaques located in CA1 or the subiculum. (**a**) Large diffuse PrP with no or negligible co-pathology with hyperphosphorylated protein tau in subject no. 4; (**b**,**c**), significant co-aggregation of PrP with bulbous neurites stained for hyperphosphorylated protein tau in subject no. 7; (**d**–**f**), and significant co-aggregation of PrP with dystrophic neurites stained for ubiquitin in subject no. 4. Primary antibodies: PrP (rabbit recombinant monoclonal antibody, green color), AT8 (mouse monoclonal antibody, red color), ubiquitin (mouse monoclonal antibody, red color). The secondary antibody was conjugated with either Alexa Fluor^®^ 488 (anti-rabbit IgG, green) or Alexa Fluor^®^ 568 (anti-mouse IgG, red). Scale bars indicate 25 µm in (**a**), and 50 µm in (**b**–**f**).

The only exception was subject no. 7, who exhibited bulbous changes in AT8-positive, bulbous dystrophic neurites in large diffuse PrP plaques in CA1 and the subiculum (Figure 5b,c). In the other subject with large PrP plaques located in CA1 and the subiculum (subject no. 4), only ubiquitin was recorded in dystrophic neurites (Figure 5d–f), but with no or negligible AT8 positivity (see Figure 5a).

### 2.3. Statistics

Primary survival data in our cohort (Table 1) and available data from the literature (Table 2 and Table 3) were analyzed relative to the presence or absence of amyloid-beta protein co-expression. None of the subjects in our cohort expressed amyloid-beta protein. The average age at death for our group was 53.78 years (±11.19 years). Data from the literature showed that in subjects with amyloid-beta protein co-expression, the average age of death was 63.92 years, which was a statistically significant difference (*p* < 0.05). Considering cases from the literature lacking Aβ-PrP co-expression, the average age of death was 52.32 years. Combining the survival data from our cohort with that from the literature, the average age of death for subjects without amyloid-beta protein co-expression was 52.74 years, which was a statistically significant difference *p* < 0.005 compared to the above-mentioned literature survival data for subjects with amyloid-beta protein co-expression.

Survival data in our study for subjects with the P102L mutation (Table 1) were also compared with data from the literature either for the P102L mutation (Table 2) or other mutations (Table 3). No statistically significant differences were found between the cohorts. The average age at death in our P102L subjects was 53.78 years compared to 52.00 years (±7.48 years) for the P102L literature group. For the P105L group from the literature, the age of death was 52.60 years and 58.67 years for the other mutations. Overall average for other than P102L mutations was 54.68 years (±10.34 years).

Statistical analysis showed a statistically significant difference of 11.18 years between the age of death in those with and without amyloid-beta protein co-expression with expectable co-expression at a later age at death. No age-related differences were observed when comparing cohorts relative to particular mutations.

**Table 1:** Summary of information for a Czech cohort of clinically, neuropathologically, and genetically confirmed Gerstmann–Sträussler–Scheinker syndrome, all having the P102L mutation in the PRNP gene.

Gender	Age of Onset	Duration	Age of Death	MV Polymorph	PRNP Mutation	Aβ-PrP Coloc.	AT8-PrP Coloc.	Others
1. F	42	1 year	43	MM	P102L	NO	YES	
2. M	65	3 months	65	MM	P102L	NO	YES	
3. M	37	2 years	39	MV	P102L	NO	YES	Son of subject no. 4
4. F	54	7 years	61	MM	P102L	NO	YES	PART; Mother of subject no. 3
5. M	61	5 months	61	MM	P102L	NO	YES	
6. M	56	5 years	61	MM	P102L	NO	YES	PART; Father of subject no. 7
7. F	29	10 years	39	MM	P102L	NO	YES	PART; Daughter of subject no. 6
8. F	NA	NA	69	MM	P102L	NO	YES	PART; M. Fahr
9. F	42	4 years	46	MM	P102L	NO	YES	FTLD-tau (PART, AGD), FTDL-TDP

Explanatory notes: NA—not available; coloc.—colocalization; PART—primary age-related tauopathy; M. Fahr—morbus Fahr/Fahr disease; FTLD-tau—frontotemporal lobar degeneration-tau; AGD—argyrophilic grain disease; FTLD-TDP—frontotemporal lobar degeneration with ubiquitin and TDP-43 positive neuronal inclusions.

**Table 2:** Summary of GSS cases having the P102L mutation in PRNP gene described in the literature with information on amyloid-beta and hyperphosphorylated tau protein colocalization with pathological prion protein including all available details. (coloc. = colocalization).

Gender	Age of Onset	Duration	Age of Death	MV Polymorph	PRNP Mutation	Aβ-PrP Coloc.	AT8-PrP Coloc.	Others, Reference
10. M	59	2 years	61	MM	P102L	NO	YES	[12]
11. F	38	7 years	55	MM	P102L	NO	YES	[12]
12. M	51	2 years	53	MM	P102L	NO	YES	[12]
13. M	59	3 years	62	MM	P102L	NO	YES	[12]
14. F	38	3 years	41	MM	P102L	NO	YES	[12]
15. M	38	6 years	44	NA	P102L	YES	YES	[13]
16. F	38	10 years	48	NA	P102L	NO	YES	[14]

**Table 3:** Summary of cases found in the literature having mutations other than P102L in the PRNP gene with information on amyloid-beta and hyperphosphorylated tau protein colocalization with pathological prion protein. (Explanatory note: NA—not available; coloc.—colocalization).

Gender	Age of Onset	Duration	Age of Death	MV Polymorph	PRNP Mutation	Aβ-PrP Coloc.	AT8-PrP Coloc.	Others
17. F	38	7 years	45	MV	P105L	NO	YES	Sister of subject no. 18 [1,15]
18. F	44	12 years	56	MV	P105L	YES	YES	Sister of subject no. 17, Mother of subject no. 19 [14,15]
19. M	47	2 years	49	MV	P105L	NO	YES	Son of subject no. 18 [10]
20. M	42	11 years	53	MV	P105L	NA	NO	Family with subject no. 21 [16]
21. F	50	8 years	58	MV	P105L	NA	YES	Family with subject no. 20 [17]
22. F	38	6 years	44	MV	P105L	NA	NO	Family with subject no. 23 [16]
23. F	44	12 years	46	MV	P105L	NA	YES	Family with subject no. 22 [16]
24. F	45	8 years	53	MV	P105L	NA	YES	[18]
25. F	48	21 years	69	MV	P105L	YES	NA	[7]
26. M	42	11 years	53	MV	P105L	NO	YES	[19]
27. M	50	20 years	70	MV	H187R	NO	YES	Father of subject no. 28 [20]
28. M	33	9 years	42	VV	H187R	NO	YES	Son of subject no. 27 [20]
29. F	61	6 years	67	VV	Y218NA117A	YES	YES	[21]
30. F	64	9 years	73	NA	A117V	YES	YES	Family with subject no. 31 [9]
31. M	33	6 years	39	NA	A117V	NO	YES	Family with subject no. 30 [9]
32. M	57	4 years	61	VV	D202N	NO	YES	[22]

**Scheme 1:**
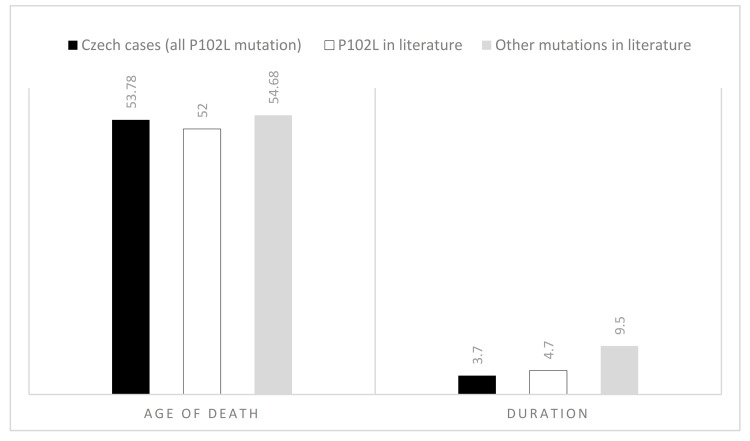
Comparison of age of death and disease duration in the cohort of Czech cases (all having P102L mutation in *PRNP* gene), cases with P102L mutation in *PRNP* from the literature, and all other mutations recorded in the literature.

The average disease durations were: 3.7 years for Czech cohort (all having P102L mutation), 4.7 years for P102L cases from the literature, and 9.5 years for cases having other mutations in the literature (see Scheme 1).

## 3. Discussion

Our results provide micromorphological and confocal immunofluorescence pictures of kuru-like plaques in a cohort of confirmed P102L GSS patients.

Contrary to our expectations, which arose from our literature review, see below, the dominant feature in the brains of our GSS subjects was condensed PrP plaques, and the majority of these structures did not show any Aβ- or tau-related co-pathology; in fact, Aβ was not recorded at all using two different antibodies (See Table 1). The condensed plaques were round, both small and large, with centrally bright immunofluorescent cores. The plaques were organized either as solitary or larger multicentric aggregates. The absence of expected co-pathologies can probably be explained by the lower toxicity of the primary PrP associated with the pathogenic mutations. It may also be related to the problematic transmissibility in specific GSS variants [23]. This “low toxicity” hypothesis is supported because GSS presents clinically from the fourth to the seventh decade of life with a relatively long disease course; it does not present in childhood [24] and not since childhood.

A minor feature in our GSS brains was the co-expression of PrP plaques with hyperphosphorylated protein tau (AT8) in dystrophic neurites. This finding is consistent with previously published data that unfortunately failed to describe the frequency [8,9]. In our cohort, there were few cases in which we observed dystrophic, tau-positive neurites co-expressing in the periphery of small solitary plaques as well as in multicentric aggregates. Large PrP plaques with prominent dystrophic neuritic changes were observed in the parahippocampal cortex of only one of the nine cases. This observation in archicortical areas could be explained by the different composition and structure of the archicortex compared to the developmentally distinct neocortex [11]. The greater tendency toward the presence of “bulbous” neuritic changes in archicortical plaques has already been demonstrated in cases with comorbid AD with Lewy body dementia or AD with amygdala predominant Lewy bodies [11].

Despite using two anti-Aβ antibodies, no co-expression of amyloid-beta in GSS PrP plaques was observed with either common immunohistochemistry or multichannel confocal fluorescence microscopy. Although the co-expression of pathological PrP and Aβ protein has been previously reported, not all subjects in the investigated cohorts exhibited this feature. Ishizava et al. reported only one subject with this co-expression in a cohort of three related patients [10], Piccardo et al. reported one subject of two [25], and Risacher et al. reported no co-expression in their only subject [26]. A detailed list of available literature related to the presence or absence of amyloid-beta protein and dystrophic neuritic changes is presented in Table 2 and Table 3. Although there are other reports, which used simultaneous double immunohistochemical staining methods to describe colocalization of PrP and Aβ [27], they failed to provide specifications regarding genetic mutations, making it impossible to compare their cases with our cohort.

The age at death seems to be a reasonable explanation for the difference between observations. Despite the fact that co-aggregation of Aβ and PrP has been proven, the relationship of Aβ presence with age suggests that development of “Alzheimer’s” pathology develops separately and independently of the PrP aggregates. The data from literature discussed above suggest that Aβ-PrP co-aggregation occurs in older subjects. In the cited articles, the average age of death was 63.92 years in subjects co-expressing Aβ and PrP, while the average age for subjects without co-expression was 52.32 years. The statistically significant difference (*p* ˂ 0.005) in the age of death between all subjects without co-expression (our cohort and literature data) compared to literature subjects with co-expression was 11.18 years (for detailed information, see Table 1, Table 2 and Table 3). In our cohort, the average age at death was 53.78 years, making the age hypothesis plausible. However, there are studies suggesting the possibility of neutralization of Aβ and PrP by mutual interaction. According to them, PrP is able to interact with both, Aβ oligomers as well as matured fibrils [28,29,30].

Even in Creutzfeldt–Jakob disease, compound PrP-Aβ plaques are not common, which agrees with our observations [4] as well as data obtained by Budka et al. [31] (only finding compound plaques in 2–29%). However, the proportion of reactive plaques in GSS that also lacked tau-positive neurites was surprisingly high, irrespective of concomitant tauopathy, namely PART and AGD, which is a frequently reported concomitant neuropathology in CJD cohorts [32].

## 4. Materials and Methods

### 4.1. Patients

A total of nine patients diagnosed with GSS (age range: 39–69 years, median age: 61 years) harboring pathogenic mutation P102L in the PRNP gene were enrolled in the study. The presence of PrP in the brain tissue was additionally confirmed using Western blot and immunohistochemistry. Patient characteristics are summarized in Table 1 and include gender, age of onset, disease duration, age of death, codon 129 methionine/valine polymorphisms, other genetic mutations, and colocalization with Aβ, and hyperphosphorylated tau protein with PrP as well as other important and/or additional information.

### 4.2. Tissue Samples

Brain tissue samples were fixed for 3–4 weeks in buffered 10% formalin. Then, using the BrainNet Europe standardized protocol [33], selected tissue blocks were embedded in paraffin using an automatic tissue processor. Sections 5 μm thick were prepared and stained with hematoxylin-eosin, Klüver–Barrera, and silver impregnation methods. For analysis, representative blocks of the left hippocampal and parahippocampal areas were chosen.

### 4.3. Immunofluorescence and Immunohistochemistry

Briefly, 5-μm-thick sections of formalin-fixed and paraffin-embedded tissue samples were deparaffinized and then incubated with primary antibodies for 20 min at room temperature. For Aβ and PrP antibody staining, 96% formic acid was applied prior to the primary antibody. A second layer for light microscopy visualization, consisting of secondary horseradish peroxidase-conjugated antibody (EnVision FLEX/HRP, Dako M822, Glostrup, Denmark), was applied for 20 min at room temperature. The samples were then incubated with DAB (Substrate—Chromogen Solution, Dako K3468, Glostrup, Denmark) for 10 min to visualize the reaction. Mayer’s Hematoxylin Solution was used as a counterstain.

For confocal microscopy, secondary antibodies conjugated to Alexa Fluor^®^ (Thermo Fischer Scientific, Waltham, MA, USA, see below) were used. Paraffin sections were also treated with 20× TrueBlack^®^ (Biotium 23007, Fremont, CA, USA) diluted in 1 × 70% alcohol to quench lipofuscin autofluorescence.

#### 4.3.1. Primary Antibodies

For immunohistochemistry, 5-µm-thick sections of formalin-fixed and paraffin-embedded tissue were selected from the left hippocampal region, including the entorhinal and transentorhinal cortex. These were incubated with primary antibodies against the following antigens: (1) Aβ (1:1000, mouse monoclonal, clone 6F/3D; Dako M0872, Glostrup, Denmark), (2) Aβ (1:5000, rabbit monoclonal, clone H31L21; Thermo Fisher Scientific 700254, Waltham, MA, USA), (3) PrP (1:8000, mouse monoclonal, clone 12F10; Bertin Pharma A03221, Bordeaux, France), (4) PrP (1:3000, mouse monoclonal, clone 6H8; Prionics 7500996, Schlieren, Switzerland), (5) PrP (1:5000, rabbit recombinant monoclonal, clone SC57-05; Thermo Fisher Scientific MA5-32202, Waltham, MA, USA), (6) Phospho-Tau (Ser202, Thr205) Monoclonal Antibody (1:500, mouse monoclonal, clone AT8; Thermo Fisher Scientific MN1020, Waltham, MA, USA), and (7) Ubiquitin (1:2000, mouse monoclonal, clone Ubi-1; MilliporeSigma MAB1510-I-25UG, Burlington, MA, USA).

#### 4.3.2. Secondary Antibodies

Detection of immunostaining was carried out using horseradish peroxidase–diaminobenzidine (see above) for immunohistochemistry and secondary antibodies conjugated with Alexa Fluor^®^ 488 (1:1000, donkey anti-rabbit, H + L IgG, Thermo Fischer Scientific, Waltham, MA, USA) and Alexa Fluor^®^ 568 (1:1000, donkey anti-mouse, H + L IgG, Thermo Fischer Scientific, Waltham, MA, USA) for immunofluorescence staining. Slides incubated with only the secondary antibody were used as specificity controls.

### 4.4. Microscopy Evaluation

#### 4.4.1. Light Microscopy

The samples were examined independently by two neuropathologists and focused predominantly on the archicortical parts of the hippocampal region; the presence or absence of Aβ deposits and AT8-positive structures, in relation to PrP deposits, was evaluated. An Olympus BX51 microscope (Olympus Europa SE and Co. KG, Hamburg, Germany) was used for examination with 100× magnification. Images were captured with an Olympus DP72 camera using Olympus image analysis software (Olympus Europa SE and Co. KG, Hamburg, Germany).

#### 4.4.2. Confocal Microscopy

Colocalization of pathogenic protein aggregates was imaged using a Leica TCS SP5 confocal fluorescent laser scanning microscope (Leica Microsystems Inc., Wetzlar, Germany). An HCX PL APO objective was used with 40× magnification, oil immersion, and a 1 AU pinhole. Donkey anti-Rabbit IgG secondary antibody was conjugated to Alexa Fluor^®^ 488 and excited at 488 nm using a 65 mW multi-line argon laser, whereas Donkey anti-Mouse IgG conjugated to Alexa Fluor^®^ 568 was excited at 561 nm using a 20 mW DPSS laser.

#### 4.4.3. Statistics

Student’s *t*-test was used for statistical analysis.

## 5. Conclusions

Despite our expectations, which came from published literature, the dominant picture in the GSS brain is small, condensed plaques that are sometimes organized into more complex plaques; however, dystrophic neuritic changes that accumulate hyperphosphorylated protein tau or amyloid-beta co-expression appear to be a minor feature and may not be related to disease development. From our results, it can be concluded that co-expression with amyloid-beta can be expected when subjects die at older ages and can probably be considered a parallel and independent age-related amyloid-beta protein pathology.

## Data Availability

The authors confirm that all data underlying the findings are fully available without restriction. All data are included within the manuscript.
